# Supplementary material for: The “Little Iron Waltz”: The Ternary Response of Paracoccidioides spp. to Iron Deprivation
Source: J Fungi (Basel). 2020 Oct 12;6(4):221. doi: 10.3390/jof6040221 (PMC7712450; doi:10.3390/jof6040221)
Supplement: Supplementary file 1 [file jof-06-00221-s001.pdf]

Supplementary material

1. Molecular dynamics

The three-dimensional structures of the MirB, MirC and Sit1 proteins of *Paracoccidioides* spp. have not been experimentally determined so far, therefore, using the I-TASSER server we performed the molecular modeling [1]. Molecular dynamics were performed using the GROMACS package, with AMBER force field (ff99SB-ILDM) and TIP3P water. The molecules were subjected to the simulation of 20 nanoseconds, temperature of 300 K, pressure of 1 atm and time interval of 2 fentoseconds. The analysis of RMSD (root-mean-square deviation of atomic positions) were performed using the software of the GROMACS package [2]. Quality scores and Ramachandran maps of proteins were performed using the MolProbity server [3].

The enterobactin and ferrioxamine B siderophores molecules were obtained from the Protein Data Bank (PDB) in the 6Q5E [4] and 4FIL [5] crystals, respectively. Carboxymicobactin was designed according to the structure described by [6]. Molecular docking was performed between siderophores and proteins using AutoDock Vina [7]. The best anchorage between the molecules, according to the energy score, was chosen for the analysis of interaction. This analysis was performed using the Discovery Studio Visualizer (BIOVIA: Dassault Systèmes, 2020), Pymol (The PyMOL Molecular Graphics System, Version 2.0 Schrödinger, LLC) and Maestro (Maestro, Schrödinger, LLC, New York, NY, 2020) software.

1.2. Results and discussion regarding molecular dynamics

In Supplementary Figure 1, the MirC and Sit1 proteins showed high mean square deviation in relation to their backbone, reaching values above 1 nm. However, the MirB protein performed better in molecular dynamics, with RMSD varying around 0.4 nm. This different behavior may be due to MirB more globular structure, while MirC and Sit1 present more regions of unstructured loops, which provide higher flexibility during the simulation. In addition, high RMSD values in the case of models generated with low similarity between crystallographic structures are expected, since there are regions without homology that were modeled by the template-free method [8].

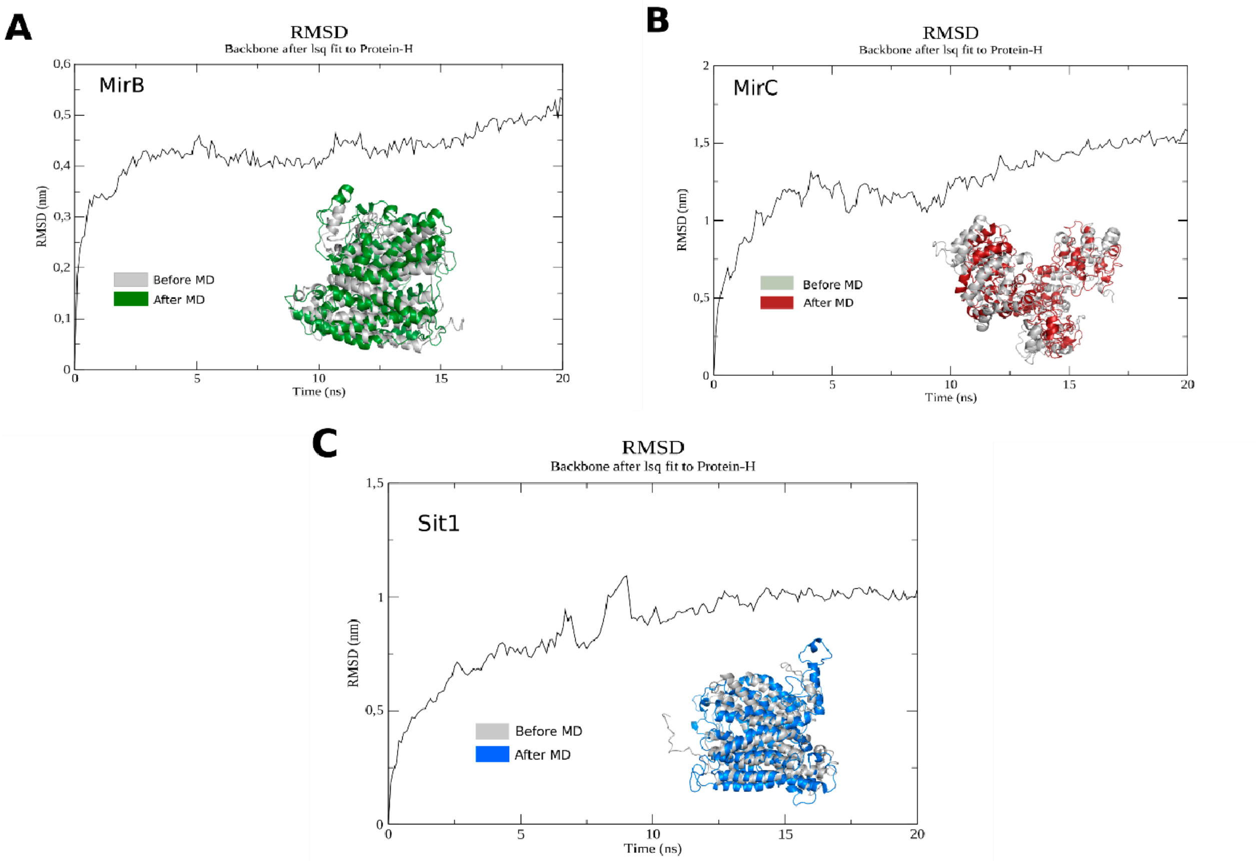

Supplementary Figure S1. RMSD in molecular dynamics simulations of the models: A) MirB showing a fluctuation between 0.4 and 0.5 nm in most of the simulation indicating in this period a more stable conformation of MirB. B) MirC showing a large fluctuation up to 1.5 nm. The Y-shaped MirB structure allowed higher flexibility and consequently higher variation of RMSD. C) Sit1 showing a fluctuation of up to 1 nm of the simulation; but it is possible to observe a stability of the RMSD from 10 ns up to the end. In all cases there is the alignment of the 3D structure, where gray is the model prior to the molecular dynamics and in green, red and blue, respectively, the most representative model of the molecular dynamics.

In molecular dynamics simulation the presence of the solvent induces the molecule to have a dynamic behavior of atoms and it is expected that it will achieve a more favorable three-dimensional structure, with adjustment of the linked and non-linked interactions [9]. In Supplementary Table 1, we note that the amino acids in favorable regions in the Ramachandran map, which refer to the Φ and Ψ angles around the alpha carbon, increased after the molecular dynamics simulation. This fact, together with the reduction of steric shocks and unfavorable interactions, made the molprobity score significantly improve. The molprobity score values achieved are comparable to those of high-quality crystal structures [3].

Supplementary Table S1: Quality of three-dimensional models after molecular dynamics simulation

	Ramachandran favored		Molprobity score	
	before MD	after MD	before MD	after MD
**MirB**	69.34%	83.65%	4.33	1.95
**MirC**	66.04%	85.05%	3.81	2.04
**Sit1**	68.98%	85.42%	4.11	1.93

In Supplementary Figures S2, S3 and S4, we observe the interaction of the carboxymycobactin, enterobactin and ferrioxamine B siderophores, respectively, with MirB, MirC and Sit1. The carboxymycobactin-MirB complex showed the interaction energy score -9.6 Kcal/mol (Supplementary Table 2), compared to the other interactions involving this siderophore. In addition to the pi-pi stacked interaction that occurs with the alignment of two aromatic rings, PHE is also involved in a special hydrogen interaction, where the aromatic ring acts as an electron donor. In this same complex, there are still 5 conventional hydrogen bonds, which are important in intermolecular recognition and in maintaining stability between molecules [10].

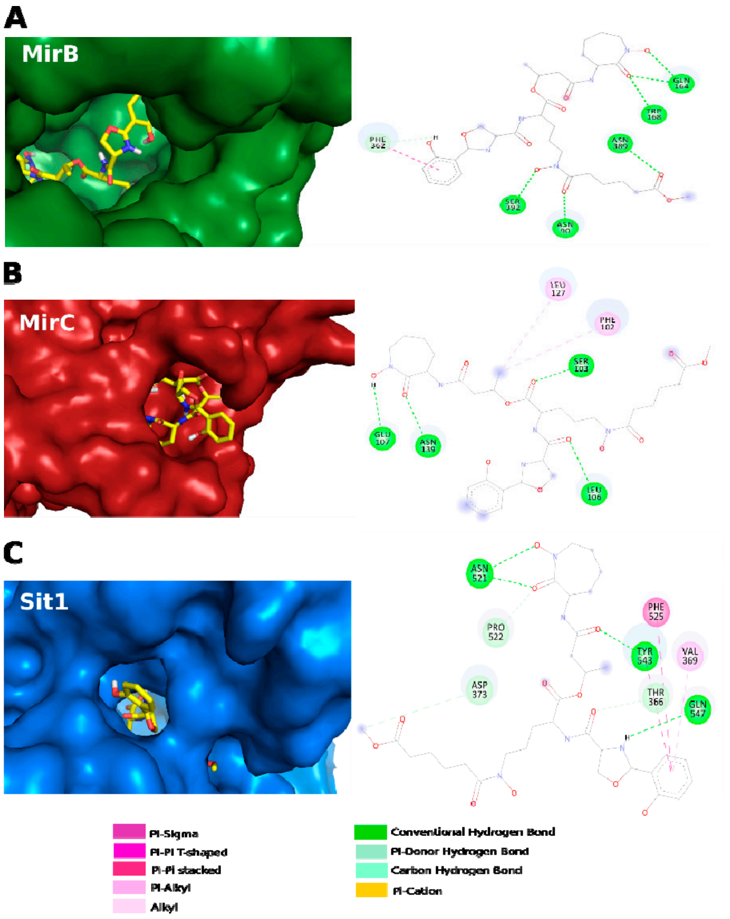

Supplementary Figure S2. 3D and 2D interactions between siderophores receptors models and carboxymycobactin.

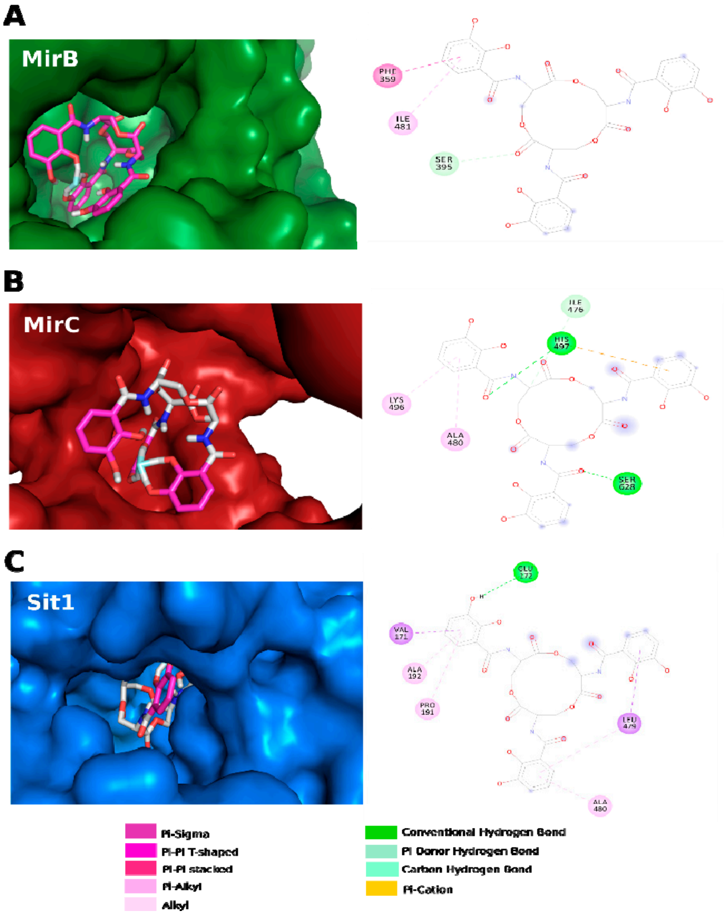

Supplementary Figure S3. 3D and 2D interactions between siderophores receptors models and enterobactin.

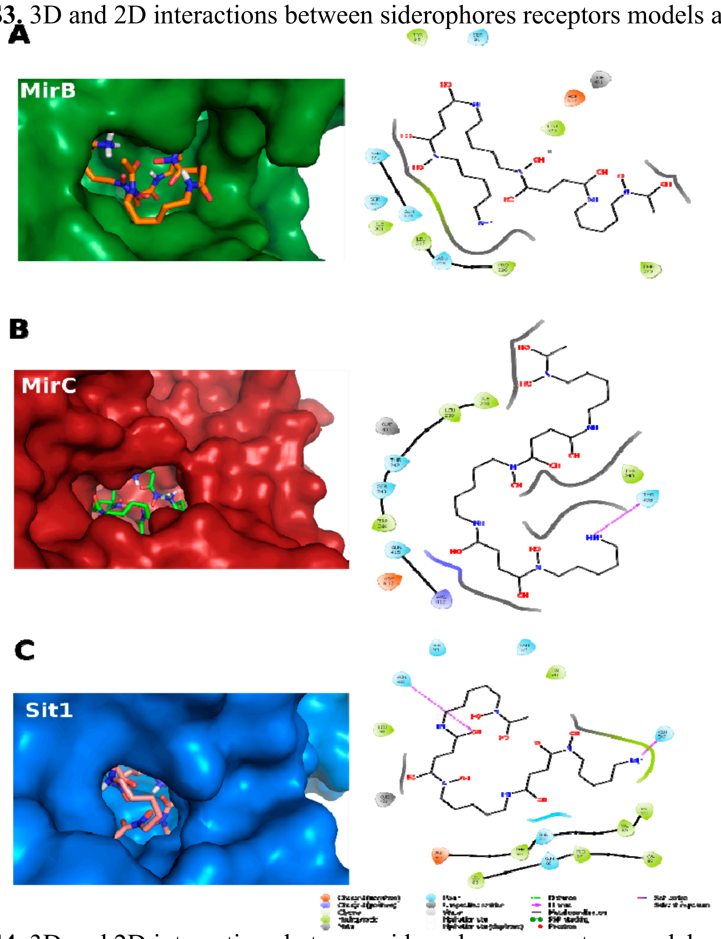

Supplementary Figure S4. 3D and 2D interactions between siderophores receptors models and ferrioxamine B.

Supplementary Table S2: Scores of binding energies between proteins and siderophores

	CMB	EB	FOB
**MirB**	-9.6	-10.7	-8.7
**MirC**	-7.1	-10.5	-8.3
**Sit1**	-8.9	-10.9	-9.8

Energy score = Kcal/mol

CMB = carboxymycobactin

EB = enterobactin

FOB = ferrioxamine B

The complexes in the case of enterobactin showed remarkably similar binding energy scores, as seen in Supplementary Table S2. In general, all complexes formed with enterobactin had several pi-type interactions, mainly because this siderophore has 3 aromatic rings in its chemical structure. It is noted that in the enterobactin-Sit1 complex, pi-alkyl and pi-sigma interactions (Supplementary Figure S3) occur that may have contributed to a slightly better energy value when compared to the other complexes of this siderophore. Among the complexes formed by ferrioxamine B, the ferrioxamine-Sit1 complex had the best energy score of -9.8 Kcal / mol. We noticed that the pocket of the Sit1 protein that interacts with FOB is highly hydrophobic which is especially important in the initial adjustment of the ligand [11].

2. **Strains and Culture Conditions**

For experiments we used yeast cells, strain 18 (ATCC32069) (P*b*18), of *P. brasiliensis*. The cells were maintained in brain heart infusion (BHI) solid medium with 4% (w/v) glucose and grown in liquid BHI for 72 hours, at 36°C. After growth to the exponential phase in liquid BHI and washes with PBS 1X (phosphate buffered saline solution 1X; 1.4 mM KH_2PO_4, 8 mM Na_2HPO_4, 140 mM NaCl, 2.7 mM KCl; pH 7.4) cells were incubated in MMcM (McVeigh/Morton medium) with no iron addition and with 50 µM of ferrous iron-specific chelator (BPS - bathophenanthroline-disulfonic acid; Sigma-Aldrich, Germany), for 24 hours at 36°C with shaking at 150 rpm. After incubation, yeast cells were collected and washed with PBS 1X. Trypan blue was used to determine the viability. For culturing under the conditions of interest was used a total of 10^7 cells/mL. Yeast cells were cultured for 6 and 24 hours in medium with 10 µM of a xenosiderophore (FOB - ferrioxamine B) and in medium with 50 µM of iron chelator BPS.

RNA extraction and quantitative real time PCR (RT-qPCR)

After incubation for 6 and 24 hours in MMcM supplemented with BPS or FOB the yeast cells were collected and total RNA extraction was accomplished using TRIzol (TRI Reagent, Sigma-Aldrich, St. Louis, MO) and mechanical cell rupture (Mini-Beadbeater – Biospec Products Inc., Bartlesville,

OK). Super-Script III Frist-Synthesis SuperMix (Invitrogen, Life Technologies) was used to obtain the cDNAs that were submitted to qRT-PCR in the StepOnePlus real-time PCR System (Applied Biosystems Inc.) using SYBER green PCR master mix (Applied Biosystems, Foster City, CA). Supplementary Table S3 features the sequences of forward and reverse oligonucleotides used. The reaction was performed in triplicate for each cDNA. Data were normalized with the transcript for 28S protein (XP_015701336) [12]. The standard curve method for relative quantification was used for calculating the relative expression levels of transcripts of interest [13]. An aliquot from each cDNA sample was used to obtain the standard curve. Statistical analysis was based in the Student's *t*-test and *P* values of 0.05 or less were considered statistically significant.

Supplementary table S3: Sequences of forward and reverse oligonucleotides

Gene	Sequence
**sit1**	F: GGCAATCATTTTCCCTGTGTG<br>R: CGCGAAGACTGCAATCAAAAG
**mirB**	F: GTCTTCTACTGGGTCGGGTAT<br>R: GACCATTCAGGAAGGCTGTC
**mirC**	F: CAGAATGTGGTGAACGCCGT<br>R: AGAATTTGCAGTCCTGTTGAAC

REFERENCES

1. Yang, J.; Zhang, Y. I-TASSER server: New development for protein structure and function predictions. *Nucleic Acids Res.* **2015**, *43*, W174–W181, doi:10.1093/nar/gkv342.
2. Pronk, S.; Páll, S.; Schulz, R.; Larsson, P.; Bjelkmar, P.; Apostolov, R.; Shirts, M.R.; Smith, J.C.; Kasson, P.M.; Van Der Spoel, D.; et al. GROMACS 4.5: A high-throughput and highly parallel open source molecular simulation toolkit. *Bioinformatics* **2013**, *29*, 845–854, doi:10.1093/bioinformatics/btt055.
3. Williams, C.J.; Headd, J.J.; Moriarty, N.W.; Prisant, M.G.; Videau, L.L.; Deis, L.N.; Verma, V.; Keedy, D.A.; Hintze, B.J.; Chen, V.B.; et al. MolProbity: More and better reference data for improved all-atom structure validation. *Protein Sci.* **2018**, *27*, 293–315, doi:10.1002/pro.3330.
4. Moynié, L.; Milenkovic, S.; Mislin, G.L.A.; Gasser, V.; Malloci, G.; Baco, E.; McCaughan, R.P.; Page, M.G.P.; Schalk, I.J.; Ceccarelli, M.; et al. The complex of ferric-enterobactin with its transporter from Pseudomonas aeruginosa suggests a two-site model. *Nat. Commun.* **2019**, *10*, 1–14, doi:10.1038/s41467-019-11508-y.
5. Podkowa, K.J.; Briere, L.A.K.; Heinrichs, D.E.; Shilton, B.H. Crystal and solution structure analysis of FhuD2 from Staphylococcus aureus in multiple Unliganded conformations and bound to ferrioxamine-B. *Biochemistry* **2014**, *53*, 2017–2031, doi:10.1021/bi401349d.
6. Agoro, R.; Mura, C. Iron supplementation therapy, a friend and foe of mycobacterial infections? *Pharmaceuticals* **2019**, *12*, 1–28, doi:10.3390/ph12020075.

7. Trott, O.; Olson, A.J. AutoDock Vina: improving the speed and accuracy of docking with a new scoring function, efficient optimization and multithreading. *J. Comput. Chem.* **2010**, *31*, 455–461, doi:10.1002/jcc.21334.

8. Carugo, O. How root-mean-square distance (r.m.s.d.) values depend on the resolution of protein structures that are compared. *J. Appl. Crystallogr.* **2003**, *36*, 125–128, doi:10.1107/S0021889802020502.

9. Karplus, M.; McCammon, J.A. Molecular dynamics simulations of biomlecules. *Nat. Struct. Biol.* **2002**, *9*, 646–652, doi:10.1038/nsb0902-646.

10. Chen, D.; Oezguen, N.; Urvil, P.; Ferguson, C.; Dann, S.M.; Savidge, T.C. Regulation of protein-ligand binding affinity by hydrogen bond pairing. *Sci. Adv.* **2016**, *2*, doi:10.1126/sciadv.1501240.

11. Wu, S.; González, M.T.; Huber, R.; Grunder, S.; Mayor, M.; Schönenberger, C.; Calame, M. Molecular junctions based on aromatic coupling. *Nat. Nanotechnol.* **2008**, *3*, 569–574, doi:10.1038/nnano.2008.237.

12. Silva, M.G.; Curcio, J.S. de; Silva-Bailão, M.G.; Lima, R.M.; Tomazett, M.V.; Souza, A.F. de; Cruz-Leite, V.R.M.; Sbaraini, N.; Bailão, A.M.; Rodrigues, F.; et al. Molecular characterization of siderophore biosynthesis in Paracoccidioides brasiliensis. *IMA Fungus* **2020**, *11*, 1–14.

13. Bookout, A.L.; Cummins, C.L.; Kramer, M.F.; Pesola, J.M.; Mangelsdorf, D.J. High-Throughput Real-Time Quantitative Reverse Transcription PCR. In *Current Protocols in Molecular Biology*; 2006; pp. 15.8.1-15.8.28.
